# Experiences of Gendered Norms and Mobilizing for Rights of Women Living With Disabilities in the Post-war Context in Sri Lanka

**DOI:** 10.3389/fsoc.2022.715240

**Published:** 2022-04-19

**Authors:** Sarala Emmanuel, Shreen Saroor

**Affiliations:** ^1^Visiting Lecturer, Department of Social Studies, Open University of Sri Lanka, Colombo, Sri Lanka; ^2^Women Action Network, Colombo, Sri Lanka

**Keywords:** gender and disability, post war and disability, disability policy and rights, Sri Lanka, post-war reparations

## Abstract

This paper juxtaposes existing legal/policy frameworks, national and international, with narratives of women living with disabilities in the post-war context in Eastern Sri Lanka. These narratives highlight their lived experience and needs. The paper draws from a process of consultations among women living with disabilities and the authors, who are long standing allies of this struggle in their capacity as activists and researchers. It focuses on key aspects of myriad gendered norms that were articulated during the consultations and describes the structural discrimination that emerges from such norms, as experienced by women living with disabilities. The paper ends with some thoughts and key proposals of women living with disabilities for the future of this struggle for justice.

## Introduction

### Vijaya^1^

Vijaya was injured during the war and her leg was amputated. She has many health complications. Every year during the rainy season her home floods resulting in more than one foot of water inside the house, and she has to manage her household work with a prosthetic leg. Her prosthetic leg needs to be repaired. Vijaya also has to go to at least three different clinics for her chronic health issues. However, she does not have the money to travel the 1 h distance by bus, from her village to the Batticaloa hospital to meet her medical needs. Vijaya was only recently included in the disability allowance scheme, even though she has been living with her injuries for more than a decade. The reasons for this delay are unknown to her despite her repeated efforts of enquiring from local government officials over the years.

As the COVID 19 pandemic spread across the country, Vijaya found it harder and harder to access any assistance. It was through calls to another woman living with disabilities in her village, who could still get around relatively more easily, that she managed to access some relief assistance.

### Saraswati

Saraswati, is a woman living with physical disabilities due to war related injuries. During the COVID-19 pandemic things became harder for her. She was in a long term, violent and abusive relationship; but when she started getting the government COVID-19 relief money of Rs. 5,000/- and also some relief goods from women's organizations, her husband became more abusive demanding the relief money and the relief items. When she refused to give him the money, he had assaulted her and forced her out of the house with the children, and they spent the night in the neighboring shrub jungle. That night, her phone battery had died she was unable to call anyone for help.

The next day, she had managed to recharge her phone from one of the neighbor's homes and called the police emergency hotline. However, they refused to come immediately because of the curfew. The police only arrived the next day in the evening, and she was taken to the hospital immediately. After she was treated for her injuries, the hospital authorities refused to allow her children to remain with her in the hospital due to COVID-19 regulations. A local women's organization made efforts to take her to a women's shelter. However, the ambulance had restrictions on where it could go as per COVID-19 regulations. She was then compelled to return to her abusive home with her children.

Vijaya's everyday struggles due to poverty, and annual monsoonal flooding, did not make her a visible priority for state assistance. Her gendered work responsibilities in the household and lack of income, meant that she was disconnected from essential health care support. Apart from the cost of the bus fare which she couldn't afford, public transport was not conducive to the needs of those living with disabilities. This further inhibited her access to the hospital. Even after approaching local state officials for assistance, during the COVID-19 context, nothing reached her.

Saraswati's experience highlights how essential services does not cover support for domestic violence during the COVID-19 pandemic. Health regulations, which are gender blind, meant that Saraswati had no choice but to go back home, as she had no place to keep her children. She had no transport services to access the women's shelter.

Drawing from the experiences of women such as Vijaya, Saraswati and many others, this paper focuses on gendered norms and structural gender discrimination experienced by women living with disabilities in the Eastern Province, Sri Lanka. It highlights the unique experiences of women living with disabilities who are at the interstices of different marginalities including, but not limited to, ethnic identity and socio-economic status. These realities exist alongside the everyday challenges to the fulfillment of their fundamental needs by virtue of systemic policies and practices that are blind to the lived realities of women living with disabilities. This paper lays bare the gendered hierarchies in the systems of assessment of disabilities and how they impact access to social welfare benefits and other basic services. Most importantly, this paper documents women's persistent efforts at collective mobilization and action as part of the struggle for their fundamental rights, to challenge gender norms and to care for one another.

## Methodology

This paper draws from a process of consultations with women living with disabilities in the Eastern Province of Sri Lanka, facilitated by the Eastern Social Development Foundation (ESDF) in Batticaloa, and led by the authors of this paper. ESDF has been mobilizing women living with disabilities in the Eastern Province for a few years and has been supporting such women with livelihood assistance and in assisting their access to state services. Through collective discussions with the leaders of the different groups of women from the three districts, the need to have a policy document with recommendations from their experiences was identified. The more the women came together and shared their experiences, the more it became clear that there was a wide gender gap in policies and services for persons living with disabilities. It was also starkly apparent that the extent of the war related impact on communities was largely hidden, including the particular experiences of women disabled by the war.

This led to a process of the authors working together with women leaders to collectively document their experiences and articulate practical recommendations that emerged from this process. These recommendations were focused on creating gender sensitive state structures that are able to meet the needs of women living with disabilities in post-war Sri Lanka.^2^

Through several focus group discussions during the months of March to July 2020,^3^ in Ampara, Batticaloa and Trincomalee- the gender norms and the discrimination thereof that mediated access to information and services were documented. Consent from members of the group for using their narratives and thoughts, anonymously in this paper was acquired at this time. The consultations also led to policy recommendations from 75 women and 11 men living with disabilities to review access to services and ensure the rights of persons living with disabilities in the post war context in Sri Lanka.

These vibrant collective processes included educating each other on UN language on disability rights; discussing language and possible translations of concepts in our own languages that gave dignity and challenged stigma; about identifying and replacing degrading terminologies in local languages; along with essential discussions on what was practical and imperative in terms of rights and services. There were two rounds of feedback integrated into the final report. One was those which were articulated in a meeting with the women leaders and another where women leaders, national level stakeholders in policy making, other disability rights organizations as well as selected state service providers were present. The original report was finalized in Sinhala, Tamil and English languages. The recommendations that came out of the consultations process was submitted to the Experts' Committee that was drafting a new constitution, under the Ministry of Justice in December 2020.

Apart from discussing gender sensitive recommendations based on the experiences of the women leaders, through the consultations. We took the time to discuss the gendered frameworks which were being put forward at the international level. The discussions were full of energy and the recommendations were clear and strong. Later, the women leaders met with the officials at the Human Rights Commission in Sri Lanka and further strengthened their recommendations. There were also many interactions and sharing of strategies with other women's groups in the North of Sri Lanka. The final submission to the constitutional committee was put together by a feminist lawyer based on these discussions. This submission was made in collaboration with three different networks—Forum of Women with Disabilities, Eastern Province; Women Organization Working on Disability, Mannar, Northern Province; and We Can—Creative Jobs for Differently Abled, Thirukketheecharam, Mannar District, Northern Province.

This paper, presented here as research findings, is one part of an already existing and continuing process of discussion and organizing of women living with disabilities along with their feminist allies who are part of the movement for the rights of women living with disabilities. It is hoped that it is one of many ways to contribute to the struggle for Justice.

## Background and Context

This paper is set in the context of post-war Sri Lanka, especially in the North and East of the island that was directly affected by the war. The war was fought primarily between the Liberation Tigers of Tamil Eelam (LTTE) whose political aim was for an independent state, and the Sri Lankan military.^4^ The war lasted for more than three decades with numerous attempts for peace negotiations, that failed. Finally, the Sri Lankan military with the support of many international actors, militarily defeated the LTTE in May 2009. During the decades of war many women had also joined the LTTE as fighters and other administrative officers.^5^ The war left deep ruptures in Sri Lankan society as a whole, also leaving a trail of destruction in the geographical regions where the fighting took place.

While the histories of the women in this paper are deeply connected to the war, it is difficult to tell their stories with a clear demarcation of “during” and “after” the war. They themselves do not speak of their journey with this differentiation. In order to remain true to their stories, they are best told when placed within a continuum that spans across this time. Similarly, the community groups of women living with disabilities, include women who were injured during the war, women who were living with disabilities from birth or other causes, and women who were carers of family members who were living with disabilities. The consultations therefore captured a broad range of gendered experiences. Thus, in the paper that follows differentiations in terms of gendered experiences of war-related disabilities and other disabilities are not made unless it was specifically narrated by the women we spoke with.

### International Policy Frameworks

Globally, there has been a shift in how disabilities have been understood and defined. Disability is no longer seen through the perspective provided by the medical model alone. People are seen as being disabled by their social contexts rather than solely by their bodies. There are two important paradigm shifts that highlight; disability as a human rights issue and disability as having a bidirectional link to poverty (Peiris-John et al., 2014; UN, 2018; UNESCAP, 2019).

The World Health Organization World Report on Disability (2011) states that “disability is an umbrella term, covering impairments, activity limitations, and participation restrictions. An impairment is a problem in bodily function or structure; an activity limitation is a difficulty encountered by an individual in executing a task or action; while a participation restriction is a problem experienced by an individual's involvement in life situations.”

The United Nations Convention on the Rights of the Persons with Disabilities (UNCRPD), was ratified by Sri Lanka in 2016. Articles 6, 16, and 28 in particular address the rights of women with disabilities and notes that State Parties are accountable “*to recognize that women and girls with disabilities are subject to multiple discrimination, and in this regard shall take measures to ensure the full and equal enjoyment by them of all human rights and fundamental freedoms and to take all appropriate measures to ensure the full development, advancement and empowerment of women, for the purpose of guaranteeing them the exercise and enjoyment of the human rights and fundamental freedoms set out in the present Convention.”* In ratifying the CRPD, Sri Lanka under international law, has an obligation to meet the requirements of this treaty.^6^ This is the global context in terms of policy from which we need to understand the domestic policy and experiences of such policies for women living with disabilities in post-war Sri Lanka (Protection of the Rights of Persons with Disabilities Act No.28, 1996).

According to Samararatne and Soldatic (2015) the existing law, The Protection of the Rights of Persons with Disabilities Act No. 28 of 1996, is not based on a rights framework and only broadly sates a “person with disability” means “any person who, as a result of any deficiency in his physical or mental capabilities, whether congenital or not, is unable by himself to ensure for himself, wholly or partly, the necessities of life”.

### Disability in Post War Sri Lanka

The Ministry of Health has noted that by 2040 the percentage of persons living with disabilities will rise to 24.4%.^7^ The reasons for this were identified as the increasing numbers of elderly and road accidents. According to a study on disability conducted by the Sri Lanka Department of Census and Statistics (2012),^8^ 1,617,924 persons live with disabilities in Sri Lanka, or 87 persons per every 1,000 persons. Among this group, 43% were male and 57% were female. It is not clear if this data even includes the war affected North and East. This data was collected in the aftermath of the war when such a process of data collection would have been difficult and any results thereof declared by the government would not be reliable.

One of the few studies conducted in the Northern Province that look into the extent of persons living with disabilities was carried out by the Ministry of Health Indigenous Medicine Probation Child Care Services Northern Province (2016).^9^ This study states that there has been a historical dearth of data on the health status of the Northern and Eastern Provinces as they were not covered by the Demographic Health Surveys. Consequently, there was little information on the prevalence of disability, despite all circumstantial evidence indicated a dramatic rise of persons living with disability due to injuries in a post-war context. According to the data collected in this report for the Killinochchi District, for example, out of 120,298 household members 13,131 persons were recorded as having a disability. This is 10.9% of those included in the study and means that one in 10 persons in Killinochchi was living with a disability.

According to a World Bank Report (2018)^10^ on the socio-economic challenges in the aftermath of the conflict, one of the groups identified as most vulnerable were ex-combatants. The report noted that around 11,000 ex-combatants had returned home. It stated that they continued to face severe challenges in finding employment along with the social stigma. The report further noted that female ex-combatants who already faced gender discrimination and profound social stigma were made further vulnerable due to them being women living with disabilities.

According to the data provided by the district offices, there were 17,487 persons registered as living with disabilities in the Eastern Province, out of which 9,461 are men and 8,026 are women. In 2019, the government of Sri Lanka increased the monthly disability allowance from Rs. 3,000/- to Rs. 5,000/-^11^ More importantly the number of beneficiaries was increased from 32,000 to 72,000^12^. This decision came about by consistent lobbying by women living with disabilities in the North of Sri Lanka with the then Minister of Finance. This show of political will by the government made a huge difference to many women as they were included in the disability allowance scheme after being on waiting lists, sometime for decades.

According to 2019 data available at the District Office 7,240 persons are registered as living with a disability in the Batticaloa District, one of the three districts in the Eastern Province^13^. However, it was not possible to get sex disaggregated data. Out of this number, 1,034 persons are receiving the disability benefit, and in 2020 this was increased to 3,442. Since then, another 2,000 forms from Batticaloa have been submitted to the Ministry of Social Services and Social Welfare, many of whom are yet to be added to the list.

## Gendered Social Norms That Mediate the Access to State Support of Women Living With Disabilities in the Eastern Province of Post-War Sri Lanka

Gender norms and patriarchal structures exert influence on all structures in Sri Lanka, be it political structures, education, economy and socio-cultural spaces. Some key indicators for this include the dismal participation of women in politics with representation of women in Parliament being at 5.3%^14^ and the low participation of women in the labor force which is at 36.6%.^15^ According to a World Bank study which analyzed data from the Household Income and Expenditure Survey (2006/7),^16^ more than 70% of women who had children were not in the labor force as they had to bear the burden of household care work.^17^ Women who step into the public realm are constantly policed by socio-cultural norms of respectability and the fear of sexual harassment. Sexual harassment is widespread in Sri Lanka with 90% of women stating that they have experienced sexual harassment in public transport.^18^ Even intimate partner violence against women is common. According to a Women's Well being Survey conducted by the Department of Census Statistics (2019) 17.4% women in Sri Lanka had experienced physical violence by a partner^19^.

Gender norms are enforced through a range of laws including the Land Development Ordinance that governs state land, which recognizes only a sole owner, namely the head of household, often enforced by the state as the male person. Even a female spouse only has life interest and no right to inheritance to this property. The eldest son is the successor. Sri Lanka also continues to have discriminatory personal laws that govern women's access to property and equal rights in the institution of marriage.

Jayawardena (2017) has shown that myths of female inferiority is used as the rationale for maintaining traditional social roles which are reinforced by the media as well as school textbooks. These range from reinforcing images and discourses that state that women are physically and mentally weaker; that women are irrational and emotional; that they are incapable of certain types of work; and the reinforcement that women's primary role is as wife and mother; that housework is women's work.^20^

These gender norms strongly influence experiences of women living with disabilities as well. These experiences deeply intersect with their experiences of ethnic identity and economic vulnerabilities in the post war context.

Manjula^*^ *had four children, and even though her oldest son lived with a physical impairment, and needed to be taken to a physiotherapist every month, she couldn't take him, as she didn't have support to leave her other three young children at home*.

Hatheesumma^*^ *had one hand amputated due a workplace injury. She broke stones for a living using one hand. Her two daughters were married and she had given them her land as dowry and now has no home to live. Her Samurdhi*^21^ *(social security) allowance was reduced as her daughters were married and were now considered as separate household units. She couldn't afford her medicines. She had applied for the disability benefit, but since her husband did not live with a disability her application was rejected. It was assumed she was his dependent and that he would provide for her as the head of household*.

Similar to the experiences of Vijaya, Saraswati, Manjula and Hatheesumma, of the 75 women we spoke with, 36% were from women-headed households and are living in poverty. Almost half of the women we spoke to, 48%, were disabled due to the war. Of those interviewed, only 50% were receiving the disability benefit, 46% were receiving other state support, while 4% were not receiving any state support at all.

All the respondents stated that their monthly income was Rs. 15,000/- or below, indicating that they were living well below the poverty line. The Poverty Head Count Index in the North and East continues to be far below the national average. According to 2016 Central Bank data, while the average Poverty Head Count Index for Sri Lanka is 4.1%, it is 11.3% for Batticaloa. Overall, it is apparent that women living with disabilities are living under extremely vulnerable conditions. These realities, unsurprisingly, conform to global thinking that there is a bi-directional link between disability and poverty.

### Gender Norms—The Household as Unit of Analysis and the Assumption of the Male Person as the Head of Household and “Provider”

One of the key manifestations of the systemic discrimination against women living with disabilities is also the very first step they must take to access state support- the disability assessment form. The Assessment Form that needs to be completed in order to qualify for the disability benefit is issued by the National Secretariat for Persons with Disabilities, which comes under the Ministry of Social Services and Social Welfare. The form and the assessment has, as its basis, the household as the unit of assessment—not the individual. A higher scoring (25) is given if the person with disability is the head of household. If it's a child or other dependent its 15 points. This means that by default, a woman living with disability who is married to a man who is not, will automatically score less even if she is the primary earner and caretaker of the family. There is no option to declare, even with proof, that she is the “head of the household.” Furthermore, a woman with disability married to a man who is not, is seen by default, to be a “dependent.” A lesser score would then mean a more arduous struggle to access the disability benefit. This makes for a reality where women and children's access to the monthly allowance is much more challenging compared with men living with disabilities.

The notion of men being the head of the household and provider of the family has a huge influence on how government officials decide who should be prioritized to get the allowance. There were experiences shared with us where disabled women and children had been asked to wait for over 4–5 years to register for their eligibility for monthly allowance. Often women's applications were rejected because of the gendered norms and assumptions that their male family members will provide for basic needs. The discriminatory nature of this assumption notwithstanding, more often than not, the male members did not provide for the rest of the family either, leaving all of them destitute.

In homes where the man and the woman both have disabilities, only the man is included in the assistance scheme. One woman shared that because her husband was disabled, she was not considered eligible for the disability assistance even though she had severe disabilities. The assumption here is that the “bread winner” of the household would automatically care and look after her. Thus, by extension she is assumed to not be the head of household when in many cases, as mentioned earlier, women in general, in this area, often are the sole earning members. This is due to a myriad of reasons which are outside the scope of this paper. These women are the sole bread winners whether they have a husband or not, irrespective of whether they are living with a disability or not.

Within the state's patriarchal system, single women living with their parents or alone are not considered deserving of state assistance. As one woman living with disabilities explained, she was told that she was not eligible for housing assistance as she was living with her parents, and she needs to marry as that is a prerequisite for her to be considered for housing assistance. Another woman shared that even the relief packs that came during the COVID-19 curfew months were not given to her by the local *Grama Niladhari* (village level government administrative officer) as they were sharing the home space with her parents. In some instances, the relief packs were reduced in amount if there were less than three persons in the household even though people living with disabilities were much more vulnerable in terms of accessing support or being able to earn an income during the COVID 19 crisis time. There was no acknowledgment that persons living with disabilities are part of the high-risk category, who are more vulnerable to illnesses and infections.

### Gendered Norms—Regulation of Women's Mobility, Presence in Public Space and Access to Information and Support

A commonly observed phenomenon in the region is the challenge faced by many women of the Islamic faith in obtaining medical records and care, as social norms necessitate the presence of a male family member to travel to the hospital with them or other districts for medical examinations. When they are unable to convince a male family member to accompany them, women often miss their clinic dates and experience delays in submitting forms for state assistance. Very similar to this, the social stigma and shame for women to be visibly disabled in public spaces, inhibit many women from accessing medical equipment and assistive devices. This further restricts their movement as such devices would have eased their mobility to some extent.

If women were to make the difficult decision to eke their livelihood through a small business, for instant, their access to loans and any other form of livelihood support is also marred by discriminatory thought and practice. Especially women living with disabilities who are head of households are assumed to be inadequate and thus a risky option in terms of providing loans. Thus, this group of women have to struggle for financial assistance in a context where they HAVE to provide not just for themselves but for the entire family.

While this challenge may be chalked up to private entities who are less accountable in general in terms of their practices from the perspective of discrimination, state services; on the other hand, do not present a much better picture. In mixed Focus Group Discussions (FGDs) with both men and women living with disabilities, it was revealed clearly that men were more aware and well informed of government assistance, programs (amendments or changes) and had details of whom to approach for this assistance. Women on the other hand did not have the information and did not know of any way to even begin to access such assistance. This is in addition to the above mentioned challenges that occurs when the woman knows how to approach any entity in the public sphere for any assistance.

### Gendered Norms—The Burden of Care Work

Abitha^*^ *is the head of her household of six members. Her husband had left her with four children many years back. She makes string hoppers for a living and earns Rs. 1,500 on a good day. She realized her youngest child had a speech impairment when he was 3 years old. She had a lot of expenses trying to make him well over the years as she began to notice that he had several developmental challenges. She took him to the government hospital, to the Mosque and also to the Samanthurai Kannakiamman Kovil (a temple of a female goddess believed to have powerful curative powers) and placed a neththi kadan (a vow) to make him well. She has now stopped the schooling of her oldest daughter so she could look after her brother. She does not get any social security benefits from the state*.

The women we spoke with strongly expressed the burden of care that is upon them to care for family members with disabilities. Children living with disabilities children are not eligible to receive the disability allowance when they are born, as the system assumes that the parents will look after them. This in turn places a double burden on the mother or grandmother who is often the primary caregiver. Apart from ensuring that the child's fundamental needs are met, they must also seek additional support for schooling, medical treatment and other resources for care from *ad hoc* sources. As noted earlier, 36% of the women we spoke with are heads of their households but are not recognized as such. Women who were caring for children with disabilities alone, mentioned that, often, they did not receive childcare support from the father of the child. Sometimes the father of their children had married other women and left the full burden of care on the mother. One Muslim woman caring for her child, said that she had been going to the Qazi court (the religio-judicial space which women of the Muslim community approach for family law matters as per the Muslim personal law) asking for maintenance, for over 2 years but to no avail.

The recommendations put forth by women living with disabilities, some of whom are also caring for children living with disabilities speaks volumes of the essential needs that remain unfulfilled. They asked for day-care centers for children and drop-in centers where persons with disabilities (particularly the elderly) could go and access recreational and learning facilities coupled with good quality care. They articulated that community-based support for persons living with disabilities should be designed to counter the over reliance on the family / household, as the burden of such over reliance is borne by women. A shared public space, where, even for a few hours, the care of persons with disabilities can be organized, would allow them to work, study, be involved in public life and also to have some rest from care-work. Such a provision even if only for a few hours was articulated as one of great relief, which stands testament to the burden they bear and the pressure they are under in their daily life.

### Gendered Hierarchies Applied to Diverse Disabilities

Saroja^*^ *was a young girl in 2009 when the war ended. She suffered from head injuries due to the aerial shelling and lost family members. She lives with shell pieces in her head and suffers intense headaches on a daily basis. She is unable to function through the day without the assistance of pain killers*.

Through conversations with women living with disabilities, it became apparent that there were hierarchies within assessment of different forms of disabilities. The Assessment Form that needs to be completed in order to qualify for the disability benefit, as mentioned earlier, is issued by the National Secretariat for Persons with Disabilities, which comes under the Ministry of Social Services and Social Welfare. The form consists of six parts. Section D of the form requires a recommendation by a medical officer. In terms of the scoring criteria to qualify for the disability benefit, it is a prerequisite that the monthly income is < Rs. 6,000/-. The highest marks, as discussed earlier, is if the person with disability is identified as the head of household, which is, by default, a male person. If an applicant has lost both hands and legs, they are awarded a score of 25. Hearing and speaking impairments get a score of 20 while the loss of one hand or one leg receives a score of 15 and so on.

Tracing the origins of this assessment for physical disabilities, scholars have turned to colonial laws that dealt with compensation for work related injuries (Campbell, 2007). This was a system of medicalized hierarchies of different values being placed on different body parts based on a notion of “productivity”; that is an overwhelming priority in terms of workers' bodies in a capitalist economy.

**Figure 1:**
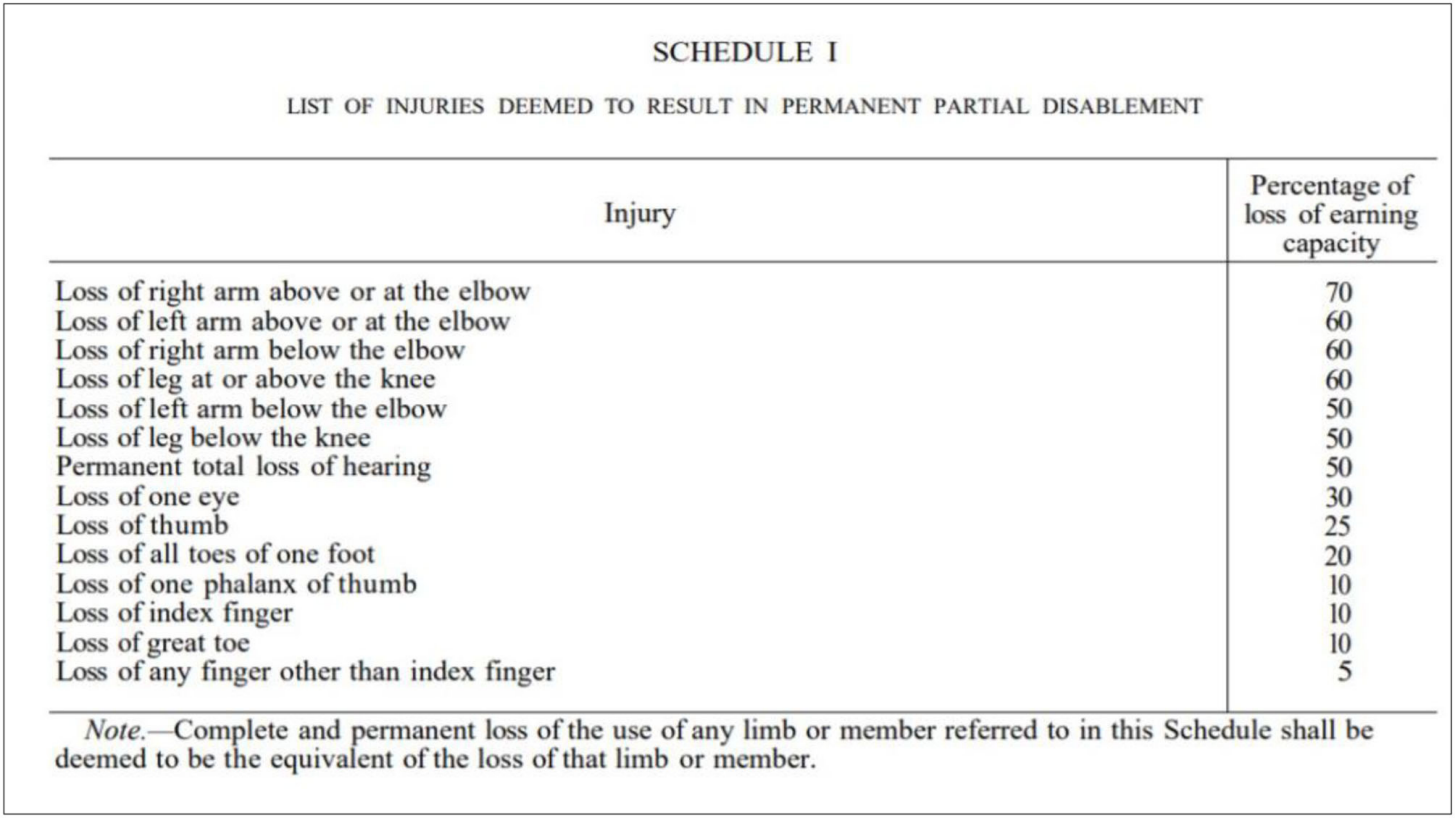
List of injuries deemed to result in permanent/partial disablement in the Workmen's Compensation Ordinance No. 19 of 1934.

The Workmen's Compensation Ordinance (1934), which still remains the law of the land, includes a definition of “partial disablement” which includes :disablement” that is temporary in nature due to an accident at work which reduces earning capacity. “Total disablement” on the other hand is one where the injury leads to no capacity for employment in any kind of work. Schedule 1 of this Act (see Figure 1) lists the percentage of loss of earning capacity based on the body part that has been disabled. This Act is important as we can clearly identify the roots of the medical report that is required for disability benefits today.

Similar to the Workman's Compensation Ordinance, the (Sri Lanka Social Security Board Act No. 17, 1996) (sections 15−17), includes a medical definition of “disablement,” which results from either an “accident” or “sickness.” Total “disablement” is usually defined as one where there is a loss of two feet or two hands, whereas partial “disablement” usually refers to a loss of one hand, foot or eye (Campbell, 2007). It is clear from these legislations that the points system for the disability benefit scheme in Sri Lanka is firmly based on existing inadequate and unempathetic medico-legal dictums of “measuring disability.”

The women who were part of our discussions repeatedly stated that the government forms did not capture their lived experience in a post-war context. The categories in the assessment form did not even have any way by which it could be recognized that their injuries were caused by war. Such recognition will fundamentally alter government accountability for such injuries, an accountability that successive regimes in Sri Lanka have evaded in general. This realm of social security and welfare on the grounds of disability is also a part of that evasion.

The data available at the District Office in Batticaloa of 7,240 persons, who are registered as living with disability, there is no sex disaggregated data. This makes it impossible to analyze this data through a gendered lens except to observe that sex-disaggregation of data, which is a rather easy task, was not even deemed to be an important priority. In the absence of such data, there has been no attempt to understand long term war related injuries and internal injuries—particularly of reproductive organs—which cause chronic pain, the inability to work and prolonged fatigue among other things. There is no space to record such conditions in the assessment form and therefore these realities are completely made invisible.

The women whom we spoke with talked about broadening the current definition of disability. Many women noted that their disability is sometimes not visible to the eyes of government officials. Even though they do not visibly use mobility related or other aids, the scars and injuries they suffer are in their vital organs. Many of these injuries are a result of the war. Their physical and mental strength has been seriously impacted by living with such disability in a society which does not enable a better standard of life for them in any manner. They noted that their inability to lead healthy lives is not considered a reason for them to be entitled to receive state support. Some of the women did not have medical records and certificates that document their disability in official parlance. Even doctors, sometimes, did not consider them as living with a disability, but as living with a “sickness” or some form of a permanent chronic condition. Chronic illness, needless to say, is seen as being beyond the scope of “disability” in the current definition. As a result, in many instances, medical professions do not certify documents which are required to gain access to the monthly disability allowance.

## Identity, Dignity, and Social Accountability as Reparations

Office of Reparations Act No. 34 (2018) is a rare legal document, in Sri Lanka, that strongly acknowledges the circumstances of persons living with disabilities. The Act notes that disability is a ground for discrimination in Sri Lanka and recognizes the continuing physical, psychosocial and economic impact. It registers the need to provide special measures including for women, children and persons living with disabilities. The Reparations Office has the mandate to design its own criteria for eligibility of aggrieved persons and decide on the form and quantity of reparations. This includes programmes for restitution, rehabilitation, welfare services, education, vocational training and psychosocial support programmes. Most importantly, it can recommend reparations which may be provided directly by other State institutions. This is significant as such possible directives from this office to other state bodies can mean an inclusion of the need for reparations and possible permeation of robust definitions of affected persons, including of those living with disabilities, more broadly in state institutions. For instance, the Reparations Act defines “aggrieved person” as follows:

“*Aggrieved person means persons who have suffered damage as a result of loss of life or damage to their person or property in the course of, consequent to, or in connection with the armed conflict which took place in the Northern and Eastern Provinces or its aftermath; such damage being in the nature of prolonged and grave damage suffered by individuals, groups or communities of people of Sri Lanka….”* (Office for Reparations Act No. 34 of 2018).

While this broad definition is a breath of fresh air in the existing law and policy regime with regards to disability in Sri Lanka, many women we spoke with had to encounter a unique challenge in obtaining medical records of war related injuries. Many of them were injured when they were combatants. If records of injuries to combatants was maintained, it would have helped to show specific war related injuries such as from shelling and bullet wounds. These records, however, do not exist anymore due to complex politically motivated loss of war-related records. Without these records, medical officers whom they approached in the post-war context, sometimes, refused to confirm these injuries citing lack of evidence. There is a general socio-political context of not documenting war-related injury beyond within the armed forces. On top of it, the women ex-combatants hold a deep fear about facing further discrimination and security threats along with the existing social stigma, if they revealed that their injuries was connected to them being combatants.

As a practical solution to this unique problem and as a way of addressing the social stigma and discrimination, women living with disabilities strongly articulated the need for Identity Cards (ID). However, as a prerequisite to issuing these ID cards, there needs to be a broadening of the definition of disability to include war related injuries and also, clear consideration of the gendered dimensions of living with disabilities in all laws and policies. As a concrete way of putting forward this demand leaders of the communities of women living with disabilities called for urgent amendment of assessment procedures to be in line with standards of the UNCRPD.

They proposed that the Office for Reparations, given its most progressive definitions with regards to “aggrieved person” and the mandate for independent definition of such persons including of those living with disability, could lead this initiative and liaise with the relevant government ministries. This Office can design the eligibility criteria and set in motion special measures to enable an ID card. Women noted the importance of this ID card for them to be treated with dignity and to enable them to access services. Many women shared the humiliating process of having to repeatedly “show” their “disability” each time they tried to access support or services. These services include negotiating special care in hospitals, a seat on the bus, while standing for long periods in a queue at the bank, while accessing livelihood assistance and most importantly, accessing any government assistance programmes for persons living with disabilities. The act of “showing disability” is a further challenge for women as often they cannot expose many parts of their bodies in largely male-dominated spaces, especially in the Sri Lankan cultural context. A special ID card would address this multipronged challenge.

In terms of process, the women suggested a state structure that was close to them—the Divisional Secretariat—to be responsible for the preparatory work. They noted that the Divisional Secretariat (DS) which is a level above the village level in terms of local governance in Sri Lanka already consists of a Disabled Peoples' Organization (DPO). They suggest that this body (DPO) could be given the task of recommending the issuance of these ID cards, which can then be certified by the DS. As the leadership of the DPO is well aware of all the local members and the veracity of their claims, the DPO is an existing structure that is an ideal choice to undertake this task. Furthermore, local level societies such as Women's Rural Development Societies, Samurdhi (Social Security Program) groups, farmers' societies, civil protection committees and school welfare societies could provide supporting letters to facilitate the ID cards. However, the ID must be officially issued in the name of a national level body, so it is applicable and accepted anywhere in Sri Lanka.

In January 2016, the Ranaviru Seva Authority (a government body for the welfare of soldiers) and the Ministry of Defense introduced an ID card called the *Virusara Privilege* in recognition of the importance of an ID card to access certain benefits and services for military and police personnel living with disabilities.^22^ This ID card allows access to medical treatment and medicines at concessionary rates. It also prevents people living with disability from having to travel without a seat (standing) in long distance buses. It allows access to housing loans, provides for children to get support for education and gives access to free bus/train passes. In February 2020, the Ministry of Defense announced it was going to issue another 200,000 of such cards within the year^23^ to military and police personnel. This card is an existing model that has already been formulated by the government, it recognizes a person living with a disability and provides access to rights and other needed provisions for such a person. These formulations has to simply be extended to ALL persons living with disabilities to enable similar access to services, easy mobility and accessibility in public spaces. This will form a basis to build lives of dignity for all persons living with disability, especially women.

## Bodily and Sexual Autonomy

Mala^*^ *was pushed by her family to have one more child as the twin children from her first pregnancy were speech impaired*.

The women who were former combatants and who returned home in the post-war context were returning to a cultural context of the gendered norms, which they had already challenged.^24^ Many faced social stigma and ostracism among those of their own community, along with security threats from the military, primarily in the form of continued surveillance. There was constant suspicion and judgment in the community of how women who were ex-combatants lived their lives.

One of the participants in our discussions succinctly explained this when she said,

“*people wonder whether we can live a normal life because of this disability and at the same time they also question our ability to lead a family life, not only because we were part of LTTE but also because now we are not ‘normal' or a ‘full woman'. ”*

Many women often heard the narrative of, “*how can she be a ‘good woman'? How is she meeting her needs and expenses? She must be flirting or having a man in her life*.” Even though some women preferred not to get married, they were compelled to do so in order to have a man in the house for what is often referred to as “protection,” which implies protection in terms of social morality rather that physical or emotional safety. A woman who was a former militant and currently lives alone stated that “*even the government officers look at us in a manner that is so sexualised, and they keep talking about us being alone*,” implying that they are “available.”

The women we spoke to strongly articulated that the decision to get married, to not get pregnant or to carry a child as well as the decision on the number of children among other things should be the woman's decision alone. They declared that a woman's bodily autonomy should be respected in every instance. They stated that in order to live independently, (including opting to not get married) they needed a community of women with support structures/spaces which were not institutionalized and run like incarceration centers. Instead, they needed spaces that treated them with dignity, autonomy and recognized their inherent right to make choices about their bodies.

## Sexual Harassment, Violence and Lack of Access to Redress

“*I went to ask for the disability allowance, but he kept asking about my husband. I live in a very abusive relationship and did not want to tell him all that, to get my allowance”*.

“*One officer asked me openly in front of other government officers—with this disability how did you manage to have three children? What does your husband do?”*

Almost all the women we spoke to, shared about the inappropriately sexualised treatment meted out to them by male government officers when they approached them for assistance.^25^ There were many incidents narrated by women describing how the officers treated them differently than men who lived with disabilities. Some spoke about how the officers' sexualised gaze itself made them not want to go back, while some reported calls late in the night “*talking about our physical attributes and sometimes asking questions that were intrusive and discomforting*.” One interviewee said “*the officers do not respect us. They do not even ask us to sit down. They ask questions loudly about our disability, that we experience it as a violation of our privacy and dignity*.”

The sexual harassment, breach of privacy and lack of dignity in the public sphere continues, including and especially in government offices which are supposed to be their safe space. Meanwhile, the home was not safe either. Similar to the experience of Saraswati, mentioned earlier, a study done by Samararatne and Soldatic (2015), noted that women living with disabilities face tremendous gender discrimination and humiliation when trying to access support for domestic violence. Many are unable to access any support or even report the violence. Those with speech impairments for example have no support to navigate any legal process.

Several women whom we spoke to mentioned that they were living in an abusive and violent home environment. However, they were unable to go to the Police or access other support services. This was because of the stigma about speaking of domestic violence in public and it adds to the general stigma they have to live with every day. Their dependance on these men for their daily support meant that they couldn't, realistically, take legal action against them. Thus, even the minimal redress available to women, in general, with regards to violence, is unavailable to women living with disabilities.

In the consultations, the women leaders recommended an independent commission for rights of persons living with disabilities. They said that such a commission should have participatory monitoring committees which are to ensure government services reach them, including those relating to domestic violence. Such a commission could also receive and act on complaints of sexual harassment and abuse while trying to access government services.

### Autonomy, Recreation, and Gendered Experiences of the Body

Veena^*^ *continued to engage in sports even with one of her legs being amputated along with her other physical injuries during the war. She is one of the few women who has been able to access the parasports competitions in Sri Lanka. She was sent to other districts (Kurunegala and Colombo) for 4 months of training, through the state services. However, she noted that she had no space nor equipment to regularly train in Batticaloa when compared to military personnel with disabilities. One of the local school principals gave her some equipment to practice. Even without any basic equipment, training gear or physical space to practice regularly, she had won nine medals in various sporting events at the national level. She also noted that for women, it was more challenging to take time off and travel in order to train in other districts, as they have child care responsibilities and also because they would have to leave their livelihood activities*.

Given this reality, women living with disabilities noted the importance of spaces that women can access for recreation, sports and physical therapy. Some of the women we spoke with were involved in parasports. However, there are no training facilities in the Eastern Province to enable them to train regularly and improve their skills. Sports, including facilities for swimming, provides important spaces for persons with disabilities to gain mental and physical well being through exercise. This is particularly important for women. Sports also helps women living with disabilities challenge social stigma and gender stereotypes and would build their self-esteem.

Even though annual parasports events are organized in Sri Lanka, it is critical that there is equal and regular access to physical spaces for sports and training sessions to improve one's skills, for all persons living with disabilities who may want to participate in these sporting events. The events in isolation are not enough as they benefit only a small group of athletes who have access to equipment, space and training. This group primarily consists of those who were in the military to whom the government has provided resources and trainers. It is important to recognize the need for similar facilities and trainers for civilians living with disabilities in the war affected North and East of Sri Lanka. Any attention paid to them will exponentially enhance the quality of life of women living with disabilities in this region.

## Conclusion

Historically, there has been some involvement of disability rights groups at the national level within law making processes in Sri Lanka. The consultations that formed the basis of this paper, which resulted in a submission to the constitutional committee, however, was the first time that strong voices of women living with disabilities in the post war context, had been put forward in regards to any national level legal and policy related process. These recommendations have inspired the conclusions of this paper as the form and content of these recommendations clearly reflect the challenges confronting women living with disabilities in post-war Sri Lanka, and the way forward to ensure their fundamental rights.

Firstly, the recommendations declare the need for a broadening of the definition of disability to include war related injuries and to reflect gendered experiences of disabilities. Secondly, it was suggested that there should be an urgent review of the assessment criteria so as to ensure that it is in line with standards of the UNCRPD. Thirdly, as two essential measures of ensuring their access to state services, they recommend the issuing of special ID cards and establishing an independent commission for rights of persons living with disabilities. Most importantly, they urge that such a commission include collective monitoring systems (with representatives of persons living with disabilities and from disability rights groups) to be put in place to ensure that government services reached all, particularly women, and to ensure that harassment and abuse are, in the least, reduced. Fourthly, in order to address the burden of care work drop-in care centers at the community level and individual support for women who are bearing the burden of care in the homes was suggested. Fifthly, as a significant measure for recreation, building strength and self-esteem, special centers for women and girls for physical therapy and sports was recommended.

We are well aware that the women leaders who were part of our consultations had been supporting/caring for each other, helping members to access services and responding to gendered discriminations/violence for many years. Through their regular collective engagement, they are documenting their stories, connecting with broader struggles such as that of women's rights/feminists, collectively being present in public spaces and in policy forums. Through this work, they are able to strengthen themselves, both; individually and collectively.

It is clear that in order for law and policy to reflect the experiences of women living with disabilities and address their needs effectively, the women themselves must take the lead in all policy related processes. While this may apply universally, in Sri Lanka, specifically, a robust recognition of the impact of the war on communities that are trying to recover in the North and East of Sri Lanka and the gendered dimensions thereof, is essential. Only then can any process of reparations begin to address the needs of the most marginalized in the post-war context. In order for this recognition to begin and it to be articulated as a priority for Sri Lanka, going forward, the society and the government must ***listen*** to the stories, challenges and astute suggestions emerging from the everyday lived realities of women living with disability.

## Author's Note

SE and SS are feminist researchers and activists who have been living and working in the North and East of Sri Lanka for over two decades. They are part of many community level women's groups who have been calling for their rights to equality and justice and have also worked extensively at national and international policy advocacy forums. The consultations organized by ESDF with women living with disabilities was funded by USAID.

## Author Contributions

Both the authors were involved in the full process of developing this policy brief. The design of methodology, literature review, consultations on recommendations was done together by both the authors. The article was also written jointly by both the authors. Both authors contributed to the article and approved the submitted version.

## Conflict of Interest

The authors declare that the research was conducted in the absence of any commercial or financial relationships that could be construed as a potential conflict of interest.

## Publisher's Note

All claims expressed in this article are solely those of the authors and do not necessarily represent those of their affiliated organizations, or those of the publisher, the editors and the reviewers. Any product that may be evaluated in this article, or claim that may be made by its manufacturer, is not guaranteed or endorsed by the publisher.

## Footnotes

^1^All names have been changed for safety.

^2^The full extensive report *Ensuring Our Rights from the Home to the Parliament: Position Paper on Gender Sensitive Service Provision and Victim-Centric Reparations for Women living with Disabilities Eastern Province (October 2020)*, was prepared by Emmanuel and Saroor (2020) in consultation with women leaders living with disabilities, facilitated by Eastern Social Development Foundation, Batticaloa. It was compiled in English, Sinhala and Tamil and published by Eastern Social Development Foundation, Batticaloa. The recommendations put forward by the women leaders were extensive and covered all aspects of their lives including, health, education, employment, transport, recreation, sports, identity, dignity, structural changes, budget allocations, support for care work, leadership and responding to violence and sexual harassment. This paper highlights a selected number of those recommendations.

^3^Due to the context of COVID-19, all FGDs and meetings carefully followed government regulations and restrictions on meetings to ensure the full safety of all those who participated in this process.

^4^The root causes for the conflict can be traced back to the emergence of the post-colonial independent Sri Lanka. During the decades of war there were numerous armed actors in the North and East, however, the LTTE was the primary militant group that became powerful. For more information also see Hoole (2016); Hoole (1990); Devotta (2005); Hoole, Rajan, UTHR(J), Sri Lanka's Colonization Experience: Development or Disaster https://uthr.org/Rajan/colonaisation.htm; http://archive.pov.org/nomoretears/timeline/; https://minorityrights.org/minorities/tamils/; https://en.wikipedia.org/wiki/Origins_of_the_Sri_Lankan_civil_war; https://www.himalmag.com/a-tale-of-two-languages/; https://www.bbc.com/news/world-south-asia-12004081

^5^International Crisis Group (2017; Paramanathan (2007); Hoole (1990); Darusman et al. (2011); Kodikara and Emmanuel (2012); Emmanuel and Kodikara (2016), and Satkunanathan (2018).

^6^Following the UN General Assembly (2017), the United Nations Department of Economic Social Affairs (2018) also recognized the challenge of gender equality and empowerment of women and girls with disabilities (Sustainable Development Goal 5) as a particular challenge to be met. That report notes that three times as many women as men had unmet healthcare needs in Sri Lanka in 2016. It also notes that in 2016, 29% of disabled persons could not get healthcare service, 12% could not afford the cost of transport to hospitals, and 15% did not have access to transport.

^7^See here for news article: https://www.newsfirst.lk/2014/02/24/disabled-persons-sri-lanka-will-increase-upto-24-2-2040/21877/

^8^Department of Census Statistics (2012).

^9^Ministry of Social Welfare (2003); Ministry of Health Indigenous Medicine Probation Child Care Services Northern Province (2016).

^10^World Bank (2018).

^11^See news article about this here: http://www.ft.lk/front-page/Monthly-allowance-for-disabled-persons-increased/44-680016.

^12^Ibid.

^13^Data print page provided to authors by local government office.

^14^World Bank Report No. 68. (2013).

^15^ibid.

^16^ibid.

^17^ibid.

^18^UNFPA (2017); Shenoy (2020).

^19^Department of Census Statistics (2019).

^20^Jayawardena (2017).

^21^Samurdhi is the Social Security Benefit Scheme given to those assessed as living below the poverty line.

^22^For more details about the *Virusara Privilege* ID card see here - http://virusara.gov.lk/en/news/201704201015-en.html and here http://virusara.gov.lk/en/special-card.html.

^23^See news article on this: http://www.adaderana.lk/news.php?nid=61040

^24^Reliefweb(2011), Sri Lanka: Fewer “I do's” for former female rebels https://reliefweb.int/report/sri-lanka/sri-lanka-fewer-i-dos-former-female-rebels; Thuppahi's blog (2021), Tamil Tigers: Unfulfilled Promises of Feminism and Liberation, https://groundviews.org/2021/02/23/tamil-tigers-unfulfilled-promises-of-feminism-and-liberation/.

^25^See also International Crisis Group (2017).
